# Twenty-seven-gauge vitrectomy for combined tractional and rhegmatogenous retinal detachment involving the macula associated with proliferative diabetic retinopathy

**DOI:** 10.1186/s40942-017-0091-x

**Published:** 2017-10-09

**Authors:** Yousef J. Cruz-Iñigo, María H. Berrocal

**Affiliations:** 1 Department of Ophthalmology, Río Piedras Medical Center, University of Puerto Rico, San Juan, 00909 Puerto Rico; 2 Berrocal & Associates, San Juan, Puerto Rico

**Keywords:** 27-Gauge vitrectomy system, Combined tractional and rhegmatogenous retinal detachments, Small gauge

## Abstract

**Background:**

To report our experience using 27-gauge pars plana vitrectomy (PPV) system for treating patients with combined tractional and rhegmatogenous retinal detachments (CTRRD) involving the macula associated with proliferative diabetic retinopathy (PDR).

**Methods:**

Retrospective noncomparative interventional cases series of 12 patients with CTRRD associated with PDR who underwent 3-port, transconjunctival 27-gauge PPV by a single surgeon. Main outcome measures were change in Snellen best corrected visual acuity (BCVA) and occurrence of intra- and post-operative complications.

**Results:**

Twelve eyes from 12 patients (9 men and 3 women) underwent 27-gauge PPV. Mean follow-up was 17 months (range 8–26 months). Preoperatively, BCVA of 20/400 or better was recorded in only 2 of 12 (16.7%) eyes. Postoperatively, BCVA improved to 20/400 or better in 11 of 12 (91.7%) eyes at 6 months (*P* = 0.001). At last follow-up, BCVA of 20/400 or better was recorded in 10 of 12 (83.3%), in comparison to 2 (16.7%) eyes at baseline (*P* = 0.004). The only intraoperative complication was an iatrogenic break in 1 eye (8.3%). Postoperative complications included vitreous hemorrhage in 4 eyes (33.3%) and transient ocular hypertension in 3 eyes (25.0%). At final follow-up anatomic success was confirmed in all eyes.

**Conclusion:**

The current study findings suggest that 27-gauge PPV is a safe and promising surgical technology for treating patients with CTRRD involving the macula associated with PDR. Smaller gauge instruments and higher cutting rates may facilitate the dissection and shaving of fibrovascular membranes, while minimizing intra- and post-operative complications.

**Electronic supplementary material:**

The online version of this article (doi:10.1186/s40942-017-0091-x) contains supplementary material, which is available to authorized users.

## Background

Transconjunctival microincision vitrectomy surgery (MIVS) rapidly gained acceptance following the introduction of 25- and 23-gauge systems. Important advantages over traditional 20-gauge surgery were faster wound healing, decreased conjunctival scarring, shorter operating time, reduced postoperative inflammation, reduced corneal astigmatism, and improved patient comfort with earlier visual recovery [1–3]. Aiming to enhance these advantages while minimizing the risk of hypotony, choroidal detachment and postoperative endophthalmitis, in 2010 Oshima et al. [4] described the use of the 27-gauge vitrectomy system. Since the introduction of this technology, only few postoperative complications have been reported including vitreous hemorrhage (VH), transient ocular hypertension and transient hypotony [4–7].

Similar to earlier experiences with small-gauge systems, surgical indications for 27-gauge pars plana vitrectomy (PPV) surgery are rapidly expanding due to its effectiveness and safety profile. Initially used for treating macular cases of lower complexity and VH [4], 27-gauge PPV surgery is now being used for more complex posterior segment cases including primary rhegmatogenous retinal detachment with or without proliferative vitreoretinopathy (PVR) and diabetic tractional retinal detachment [5, 7]. However, recent studies reporting surgical outcomes for combined tractional and rhegmatogenous detachment (CTRRD) associated to proliferative diabetic retinopathy (PDR) are limited to 20- or 23-gauge systems. These cases are considered one of the most complex diabetic surgeries as they require difficult techniques for removing fibrovascular tissue from the detached retina, closing retinal breaks and reattaching the retina while avoiding iatrogenic breaks. In the current study the experience using 27-gauge PPV system is reported for treating patients with CTRRD involving the macula associated with PDR.

## Methods

This study is a retrospective, noncomparative, interventional case series. The medical records were reviewed for all patients who underwent 3-port, transconjunctival 27-gauge PPV for CTRRD secondary to PDR between January 1, 2014 and December 31, 2016. Patients were excluded if larger gauge equivalents were used or if the retinal detachment was not combined. Eyes undergoing concurrent cataract extraction were included.

All surgical procedures were performed by one surgeon (M.H.B.) under local anesthesia using the Constellation Vitrectomy 27+ Total Plus Pak vitrectomy system **(**Alcon Laboratories, Fort Worth, Texas, USA). Cannulas were inserted in the inferotemporal, superotemporal, and superonasal quadrants 3.0–4 mm posterior to the limbus. The conjunctiva and Tenon capsule were displaced over the sclera to avoid communication between the conjunctival and scleral entry sites. Trocar cannulas were inserted with an angled (less than 90 degrees to the sclera) approach. Core vitrectomy was performed in all cases using a cut rate of 7500 cuts per minute (cpm) and linear aspiration of 0–650 mmHg. Removal of all vitreous opacities and posterior hyaloid was then performed without the need of adjuvant intravitreal triamcinolone acetonide. Areas of fibrovascular proliferation were segmented and dissected with the 27-gauge vitrectomy probe. This was followed by panretinal photocoagulation with an endophotocoagulation probe. Fluid-air exchange, fluid-gas exchange (12–16% perfluoropropane [C_3_F_8_] or 16–18% sulfur hexafluoride [SF_6_]), or silicone oil (SO) tamponade (1000 centistokes) were performed as needed. At the conclusion of each case, a peripheral retinal examination was performed with scleral depression and wide-field view to evaluate for retinal breaks. All sclerotomy sites were inspected after removal or cannulas.

Patient records were reviewed and the following data were collected: age, gender, past ocular history. In addition, the following pre- and post-operative findings were recorded: Snellen best corrected visual acuity (BCVA), intraocular pressure (IOP) measured with Goldmann applanation tonometer, slit-lamp biomicrosopy of anterior segment, fundoscopy and spectral-domain optical coherence tomography (OCT; Spectralis HRA + OCT; Heidelberg Engineering, Heidelberg, Germany). Postoperative evaluation and recording of findings were performed at 1-day, 1-week, 1-months, 3-months, 6-months, 12-months and, thereafter, at all subsequent follow-up visits. Preoperative surgical details were recorded including injection of intravitreal bevacizumab (IVB) of 12.5 mg within 3–5 days prior to surgery. Also recorded, were intraoperative surgical details including use of air, gas, or SO tamponade (1000 centistoke); and total surgical time. Intraoperative complications including bleeding, iatrogenic retinal breaks, sclerotomy site leakage and suturing were also recorded. Postoperative complications including ocular hypertension, hypotony, VH, endophthalmitis, retinal and/or choroidal detachment were documented if present. VH was classified as early postoperative (VH fewer than 6 weeks from surgery but not present on postoperative day 1), delayed postoperative (VH 6 weeks or more from surgery), or severe persistent (nonclearing for more than 6 weeks and present since postoperative day 1). Ocular hypertension was defined as an IOP of 25 mmHg or more at any visit while hypotony was defined as an IOP of 6 mmHg or less. The primary outcomes measures were change in BCVA, retinal re-attachment and presence of intra- and post-operative complications.

## Results

Twelve eyes from 12 patients (9 men and 3 women) with history of PDR secondary to type 2 diabetes mellitus were referred to our clinic due to CTRRD involving the macula (Table 1). Mean age at first evaluation and surgical intervention was 53 years (standard deviation 7 years, range 38–72 years). Mean follow-up was 17 months (range 8–26 months). Ocular history was only relevant for pseudophakia in 3 eyes. Remaining 9 eyes were phakic. At first evaluation, funduscopic exam and macular OCT revealed broad fibrovascular membranes along the arcades with adhesions in more than 2 sites causing a TRD with associated rhegmatogenous retinal detachment in all cases.

**Table 1:** Baseline characteristics, pre- and post-operative visual acuity outcomes following 27-gauge pars plana vitrectomy for combined tractional and rhegmatogenous retinal detachment

Case	Age (years)	Gender	Duration of follow-up (months)	Best-corrected visual acuity		
				Pre-operative	Post-operative	
					6 month	Last follow-up
1	54	Female	20	5/200	20/400	20/400
2	60	Male	15	20/100	20/50	20/50
3	58	Male	25	4/200	1/200	HM
4	48	Male	26	3/200	20/50	20/40
5	64	Male	26	2/200	20/50	20/70
6	53	Male	16	3/200	20/400	20/400
7	50	Female	12	5/200	20/100	20/80
8	54	Male	8	20/200	20/100	20/100
9	60	Male	13	5/200	20/400	20/400
10	52	Male	18	1/200	20/400	20/400
11	39	Male	13	HM	20/400	3/200
12	49	Female	18	2/200	20/200	20/200

Adjuvant preoperative IVB was used in all cases. All patients underwent 27-gauge PPV surgery without the need conversion to a larger vitrectomy system or using bimanual techniques. Only, one case underwent concurrent cataract extraction and intraocular lens implantation at the time of vitrectomy. In addition, one case required temporary intraoperative tamponade with perfluorocarbon liquid to help flattened the retina (patient 2 in Table 1). All cases were completed without the need of performing retinotomies or retinectomies. Mean operative time was 50 min (standard deviation 5 min, range 45–58 min). The only intraoperative complication was an iatrogenic break during removal of fibrovascular membranes in 1 eye, requiring silicone oil tamponade (patient 1 in Table 1). The remaining 11 eyes were left with a gas-filled vitreous cavity, 10 eyes with 17% C_3_F_8_ and 1 eye with 25% SF_6_. All sclerotomies were self-sealed without the need of suture placement.

BCVA outcomes are shown in Table 1. Preoperatively, BCVA of 20/400 or better was recorded in only 2 of 12 (16.7%) eyes. Postoperatively, BCVA improved to 20/400 or better in 11 of 12 (91.7%) eyes at 6 months (*P* = 0.001). At last follow-up, BCVA of 20/400 or better was recorded in 10 of 12 (83.3%), in comparison to 2 (16.7%) eyes at baseline (*P* = 0.004).

Postoperative complications included VH in 4 eyes (33.3%), transient ocular hypertension in 3 eyes (25.0%), and retinal re-detachment in 1 of the eyes with VH (patient 3 in Table 1). All cases of postoperative VH were classified as early and resolved within the first month without the need for reoperation. The patient with the detachment achieved anatomic re-attachment following PPV with silicone oil tamponade. Transient ocular hypertension was during the first week in 3 eyes (25.0%, range 25–36 mmHg), all of which responded promptly to topical treatment with brinzolamide (1.0%) every 8 h.

**Fig. 1:**
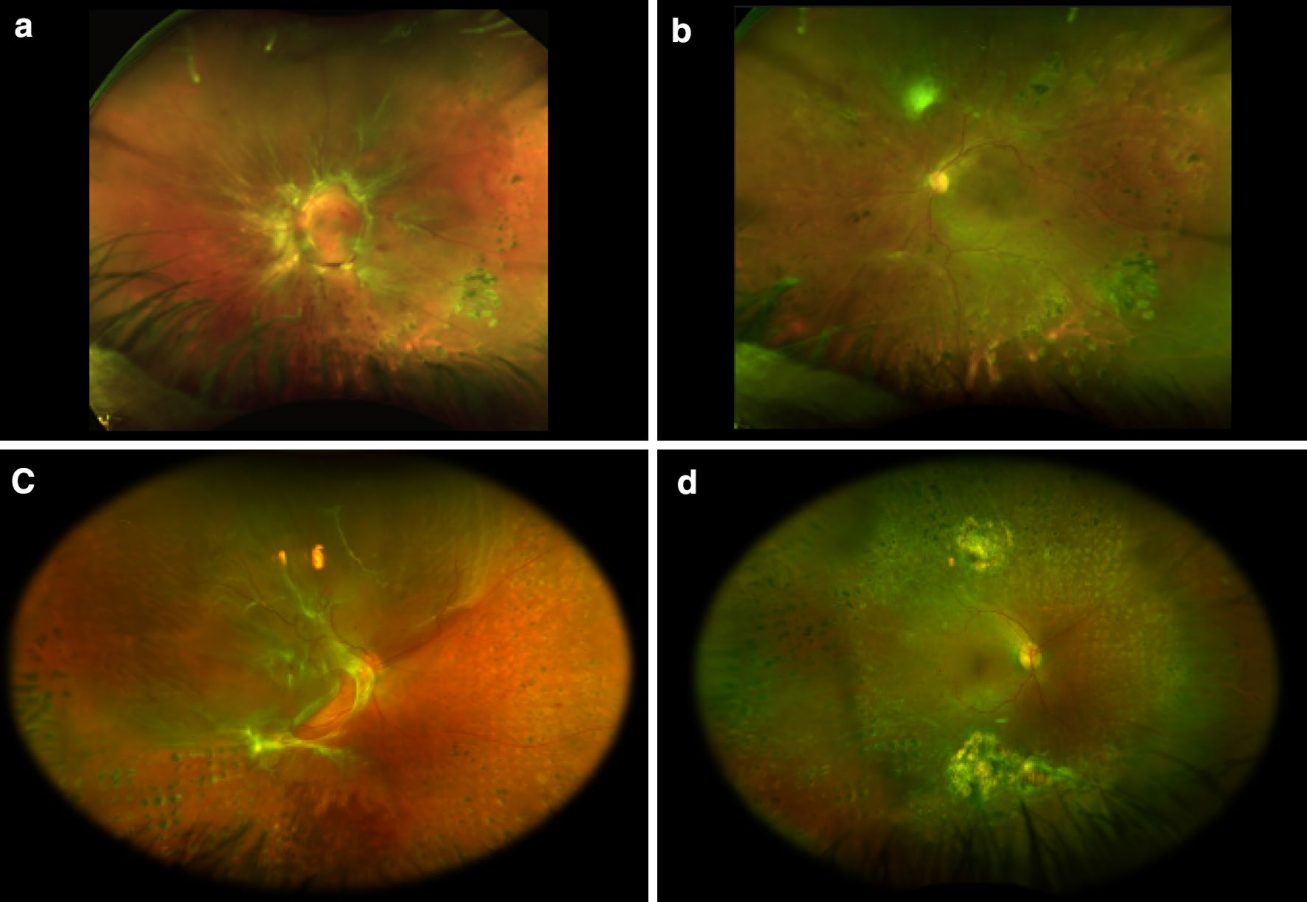
Case 4 (left eye); **a** Preoperative ultra-widefield fundus photograph showed combined tractional and rhegmatogenous retinal detachment with fibrovascular proliferation along the arcades; **b** postoperative fundus ultra-widefield fundus photograph showed retinal attachment with six months after surgery. Case 7 (right eye); **c** preoperative ultra-widefield fundus photograph showed combined tractional and rhegmatogenous retinal detachment; **d** postoperative fundus ultra-widefield fundus photograph showed retinal attachment 1 year after surgery

At final follow-up anatomic re-attachment was confirmed in all eyes. Examples of pre- and post-operative ultra-widefield fundus photographs are shown in Fig. 1. In addition, two surgical video files are included as online-only material (Additional file 1 and Additional file 2).

## Discussion

The results of the current study suggest that 27-gauge MIVS system is a useful surgical alternative for the management of CTRRD as minimal intraoperative complications occurred and BCVA remained stable or improved in 91.6% of eyes. Postoperative complications included VH in 33.3% of eyes and transient ocular hypertension in 25.0%. Only one patient required a subsequent surgery. At final follow-up anatomic success was confirmed in all eyes.

At present, studies reporting on surgical techniques and outcomes for treating CTRRD include 20-, 23- and 25-gauge systems [8–11]. In 2003, Douglas et al. [8] evaluated the surgical outcomes of 22 eyes with CTRRD who underwent 20-gauge pars plana lensectomy, PPV and SO tamponade. Within one month, 13.6% of eyes developed IOP ≥35 mmHg resulting from silicone oil migration to anterior chamber. At 6 months postoperatively, BCVA worsened in 27% of eyes. The rate of reoperation for either epiretinal membrane formation or recurrent RD was 63.6%. Subsequently, in 2008 Yang et al. [9] evaluated the surgical outcomes of 40 eyes with CTRRD who underwent 20-gauge PPV with scleral buckle and, in selected cases, SO tamponade was also used. After 6 month follow-up period, BCVA decreased in 15% of eyes and the rate of recurrent RD requiring reoperation was 7.5%. Then in 2011, Farouk et al. [10] evaluated the surgical outcomes of 200 eyes with PDR, including 6 eyes with CTTRD. All surgeries were performed using 25-gauge PPV and, in selected cases, either intraocular tamponade with air, C_3_F_8_ or SO were used. Sub-analysis limited to patients with CTTRD was only performed to assess for anatomic success 6 months after surgical intervention. In this study, the reoperation rate for CTTRD was 17.0%. More recently, in 2014 Hsu et al. [11] evaluated the surgical outcomes in 57 eyes with CTRRD who underwent 20- or 23-gauge vitrectomy with IVB at the end of surgery. In selected cases one or more of the following surgical strategies were used: pre-operative IVB, scleral buckle, and, gas or SO tamponade. At a mean follow-up of 24.4 months, BCVA worsened in 7.0% of eyes, the rate of recurrent RD requiring reoperation was 7.0% and recurrent VH was seen in 3.5% of eyes. In these studies operative time and intraoperative complications were not reported, while in the latter two studies IOP results were not addressed.

In the current study the only intraoperative complication noted was an iatrogenic break in the already detached retina in 1 eye, while postoperative complications included VH, transient ocular hypertension and a recurrent retinal detachment. Similar to the current study findings, other studies reporting surgical outcomes using the 27-gauge PPV system for treating various vitreous, macular and other vitreoretinal diseases have reported no intraoperative complications. Only few postoperative complications have been reported [4–7]. In 2010, Oshima et al. [4] first evaluated the efficiency, safety and feasibility of 27-gauge MIVS in 31 eyes with a variety of vitreoretinal diseases and found that recurrent VH was the only postoperative complication, only observed in 2 eyes (6.5%). In 2015 Rizzo et al. [6] evaluated the surgical outcomes of 16 eyes with a variety of posterior segment diseases, and found no postoperative complications. More recently, Khan et al. [7] evaluated the surgical outcomes of 27-gauge vitrectomy in 95 eyes with a variety of posterior segment diseases, and found that postoperative complications were limited to recurrent retinal detachment in 10% of cases with RD, transient ocular hypertension in 8% of all cases, transient hypotony and VH occuring in 5% each. Similar to these studies [4, 5], postoperative VH also resolved spontaneously in our study without the need of additional surgical intervention. Because recurrent bleeding in PDR is a well-known finding postoperatively [11, 12], we agree with Oshima et al. [4] that VH in our series could be attributed to our patients’ history of PDR rather than the surgical technique used.

In the current series, no cases of significant intraoperative bleeding occurred. Postoperatively, VH occurred in 33.3% of cases, all of which were classified as early and none required reoperation. Other studies have reported postoperative VH following PPV for PDR in 12–63% of cases of which approximately one-third required a second vitrectomy [12–19]. Associated risk factors for postoperative VH include incomplete preoperative scatter laser photocoagulation, active fibrovascular tissue, phakia, younger age, and hypotony [19–21]. In the current study, all patients who developed early postoperative VH were phakic, showed active fibrovascular membranes and none experienced ocular hypotony. Preoperative panretinal photocoagulation was not performed due to the risk of contraction of preretinal membranes [22–24], instead it was performed at end of surgery. To reduce the risk of intraoperative bleeding, IVB was injected 3–5 days prior to surgery in this series, but no more than 5 days to avoid causing fibrovascular contraction and subsequent worsening of the retinal detachment [25–27]. Although the evidence suggests that perioperative IVB helps prevent intraoperative VH [28–32], the evidence is contradictory for early postoperative VH [33, 34]. Similar to Khuthaila et al. [19] the authors of the current study agree that early postoperative VH was likely secondary to more aggressive PDR. The absence of late postoperative VH suggest that 27-gauge PPV with adjuvant IVB facilitated the complete dissection of fibrovascular membranes and that intraoperative panretinal photocoagulation helped achieved regression of neovascularization following surgery.

CTRRD is considered one of the most visually compromising and surgically challenging forms of retinal detachment. Surgical complexity results from severe ocular ischemia causing retinal atrophy and thinness predisposing to iatrogenic breaks [35, 36]. In the current study, use of the 27-gauge PPV system provided important advantages for avoiding iatrogenic breaks while facilitating removal of fibrovascular tissue from the detached retina, closing retinal breaks, and reattaching the retina. Prior studies have shown that tractional forces are reduced with MIVS [37, 38]. Particularly, Dugel et al. [37] showed that the enhanced 27-gauge probe had the shortest attraction across all cutting speeds and duty cycles followed by the enhanced 25-gauge and 23-gauge probes. Also, the 27-gauge technology provides additional advantages including the ultrahigh-speed vitreous cutter (7500 cuts/min) with duty cycle controlled by dual pneumatic cutters [39, 40], resulting in smaller bit size while simultaneously decreasing vitreous viscosity and maintaining efficient flow rate (Abulon DJK, et al. IOVS 2012;53:ARVOE-Abstract36915). Although some concerns regarding the decreased rigidity and lack of dual functionality of the instrumentation have been noted, in our experience the small 27-gauge vitreous cutter introduced between the membranes to dissect and shave the fibrovascular tissue is ideal for treating CTTRD while avoiding iatrogenic breaks.

Limitations of the current study include the small sample size, the lack of a control group undergoing similar surgery with a larger gauge instrumentation and the absence of preoperative fluorescein angiography results. The small sample size could raise concerns regarding the reproducibility of results. However, published studies using 27-gauge technology for treating other complex vitreoretinal cases have reported similar favorable outcomes with only few complications [5, 7]. In addition, the occurrence of CTRRD is declining as diabetic retinopathy is being treated earlier with anti-VEGF agents [41]. Likely, this will limit future studies sample size while making results from smaller sample studies more valuable. In addition, due to the lack of a control group undergoing similar surgery with a large gauge instrumentation, the current study cannot assess the efficacy of 27-gauge MIVS. Finally, routine preoperative fluorescein angiography was not performed. As a result, macular ischemia prior to surgical intervention could not be addressed.

In summary, results of the current study suggest that 27-gauge PPV is a safe and promising surgical technology for treating patients with CTRRD involving the macula associated with PDR. Smaller gauge instruments and higher cutting rates may facilitate the dissection and shaving of fibrovascular membranes, and also may minimize intra- and post-operative complications. Future studies comparing surgical outcomes with larger gauge equivalents for treating CTTRD are needed to assess the advantages and limitations of this technology.

## Additional files

**Additional file 1.** Surgical video of twenty-seven-gauge vitrectomy for dissecting and shaving fibrovascular tissue in a patient with a combined tractional and rhegmatogenous retinal detachment. Also, shown is this video is removal of subretinal fibrosis.

**Additional file 2.** Surgical video of twenty-seven-gauge vitrectomy for dissecting and shaving fibrovascular tissue in a patient with a combined tractional and rhegmatogenous retinal detachment.

## Abbreviations

PPV: pars plana vitrectomy
CTRRD: combined tractional and rhegmatogenous retinal detachments
BCVA: best corrected visual acuity
PDR: proliferative diabetic retinopathy
MIVS: transconjunctival microincision vitrectomy surgery
VH: vitreous hemorrhage
SF_6_: sulfur hexafluoride
C_3_F_8_: perfluoropropane
SO: silicone oil
IOP: intraocular pressure
OCT: optical coherence tomography
IVB: intravitreal bevacizumab
